# Hypoxia-mediated YTHDF2 overexpression promotes lung squamous cell carcinoma progression by activation of the mTOR/AKT axis

**DOI:** 10.1186/s12935-021-02368-y

**Published:** 2022-01-07

**Authors:** Peng Xu, Kang Hu, Ping Zhang, Zhi-Gang Sun, Nan Zhang

**Affiliations:** 1 Cheeloo College of Medicine, Shandong University, Jinan, 250013 Shangdong China; 2 School of Clinical Medicine, Weifang Medical University, Weifang, 261053 Shangdong China; 3 Department of Thoracic Surgery, Jinan Central Hospital, Cheeloo College of Medicine, Shandong University, Jinan, 250013 Shangdong China; 4 Department of Oncology, Jinan Central Hospital, Cheeloo College of Medicine, Shandong University, Jinan, 250013 Shangdong China

**Keywords:** YTHDF2, Hypoxia, mTOR/AKT, EMT, METTL14, LUSC

## Abstract

**Background:**

N6-methyladenosine (m6A) is a dynamic and reversible internal RNA structure of eukaryotic mRNA. YTH domain family 2 (YTHDF2), an m6A-specific reader YTH domain family, plays fundamental roles in several types of cancer. However, the function of YTHDF2 in lung squamous cell carcinoma (LUSC) remains elusive.

**Methods:**

The knockdown and overexpression of YTHDF2 in LUSC cells were conducted to detect the biological characteristics of YTHDF2. In vivo assays, the role of YTHDF2 in tumor growth was further uncovered. In vitro assays, YTHDF2 was confirmed to be involved in activating the mTOR/AKT signaling and YTHDF2 overexpression induced the EMT process in LUSC. Clinically, immunohistochemical staining revealed the relationship between YTHDF2 expression levels and the clinicopathological characteristics of lung squamous cell carcinoma patients. Moreover, quantitative PCR (qPCR), western blot, CCK8 assay, transwell assay, and wound-healing assay were used to detect the expression level and function of YTHDF2 under hypoxia exposure in LUSC cells.

**Results:**

The results showed that hypoxia-mediated YTHDF2 overexpression promotes cell proliferation and invasion by activating the mTOR/AKT axis, and YTHDF2 overexpression induces the EMT process in LUSC. Moreover, YTHDF2 is closely associated with pN (pN– 37.0%, pN + 73.9%; P = 0.002) and pTNM stage (pI 50.0%, PII 43.3%, pIIIa 80.6%; P = 0.007), ultimately resulting in poor survival for LUSC patients.

**Conclusion:**

In brief, the results highlight high-YTHDF2 expression predicted a worse prognosis of LUSC, while hypoxia-mediated YTHDF2 overexpression promotes lung squamous cell carcinoma progression by activation of the mTOR/AKT signaling pathway.

**Supplementary Information:**

The online version contains supplementary material available at 10.1186/s12935-021-02368-y.

## Introduction

Worldwide, malignant tumors of the lung are the primary cause of cancer incidence and death, ranking as the highest tumor-related mortality with more than 1.8 million deaths in 2018 and accounting for almost 1 in 5 cancer deaths [1]. Lung squamous cell carcinoma (LUSC) and lung adenocarcinoma (LUAD) are the most common histological subtypes of non-small cell lung cancer (NSCLC), which accounts for almost 80%–85% of all human lung cancers. Targeted drugs for specific gene mutations have recently greatly improved the clinical prognosis of advanced LUAD patients. In contrast, LUSC patients have a poor clinical prognosis and lack targeted agents compared to LUAD patients [2–4]. Therefore, there is an urgent need to search for new oncogenic drivers to inhibit the development and progression of LUSC patients.

The cellular response to hypoxia, followed by activation of hypoxia-inducible factor 1 (HIF-1), has been reported to be emerging as an important mechanism promoting tumor aggressiveness, metastasis, and poor prognosis [5]. N6-methyadenosine (m6A), the most prevalent modification of mRNA, is not only induced by hypoxia and promotes cancer progression, angiogenesis, and metastasis in several cancers [6, 7], but is also widely involved in many biological processes, such as splicing and stability of mRNA, RNA nucleation, the interaction between RNA and protein, and protein translation [8–10]. To date, hypoxia, as an attractive therapeutic target, has not been successfully exploited in the lung [11]. Consequently, it is critical to further investigate the molecular mechanism of hypoxia exposure and the prognosis of lung squamous cell carcinoma.

In addition, m6A RNA modification acts as a dynamic and reversible internal RNA modification process promoted by a ‘writer’ complex (METTL3, METTL14, WTAP, and other undiscovered subunits), inhibited by ‘erasers’ (FTO and ALKBH5), and functionally executed by ‘readers’ (the YTH domain-containing family (YTHDF1-3, YTHDC1-2) and HNRNP family proteins) [12–14]. Studies documenting the “reader” proteins indicate that they mainly mediate the fates of m6A modified mRNAs, such as mRNA processing, translation and degradation, microRNA (miRNA) processing, and nuclear export [8, 15–17]. Recognition by the IGF2BP family can enhance the target mRNA stability [18]. m6A-containing transcript translation was promoted by a combination of YTHDF1 and YTHDF3 [19–21]. Furthermore, YTHDF2 could regulate m6A-modified mRNA degradation [10]. In summary, the biological functions of m6A modification in mRNA have been reported to contribute to regulating the progression of cancer development [22–24].

More than 90% of cancer-related mortality is associated with cancer cell metastasis [25]. Epithelial-mesenchymal transition (EMT) plays an important function in cell migration, invasion, and cancer progression, endowing cells with stem cell properties and contributing to immunosuppression [26]. Furthermore, Snail superfamily members, the prominent inducers in EMT, are very strongly implicated in tumor grade, recurrence, metastasis, and poor prognosis in various tumors types [27, 28]. Recently, it has been reported that RNA epigenetic factors mediate EMT progression and the development of cancer [29]. For example, adverse prognosis in liver patients was associated with the coregulation of METTL3 and YTHDF1. Moreover, studies highlight the key role of m6A in EMT progression, cancer metastasis, and YTHDF1-mediated Snail translation [30]. The roles of m6A in the EMT process and Snail expression need to be further investigated in other cancers. Certainly, our findings indicated that upregulation of YTHDF2 induces the EMT process, and predicts a worse prognosis in LUSC patients.

It has been reported that YTHDF2 expression is promoted in multiple tumors including lung cancer patients [31–33]. In this study, we further explored the expression level and biological role of YTHDF2 in lung squamous cell cancer. Our data showed that YTHDF2 was mediated by hypoxia exposure and orchestrated proliferation and invasion in lung squamous cell cancer. Mechanistically, hypoxia-stimulated HIF-dependent upregulation of YTHDF2 activated mTOR/AKT signaling pathway. Thus, we hope that this study can further explore the molecular mechanism of hypoxia exposure and provide potential therapeutic targets for LUSC.

## Methods

### Cell culture

In a humidified incubator (37℃, 5% CO2), NCI-H226 and SK-MES-1 cells from the American Tissue Culture Collection (ATCC), were cultured in DMEM medium (KeyGEN BioTECH, Nanjing, China), and 10% fetal bovine serum (Gibco, United Kingdom) and 1% penicillin–streptomycin solution (KeyGEN BioTECH, Nanjing, China) were added. Using shRNA lentiviral particles (GENECHEM, Shanghai, China) containing YTHDF2 and the control pNull (empty vector), two cell lines were infected according to the infection instructions to achieve the stable upregulation of exogenous YTHDF2. The upregulation of exogenous protein expression was detected by western blotting. The YTHDF2-KD were amplified by PCR and cloned into hU6-MCS-Ubiquitin-mCherry-IRES-Neomycin vector (GENECHEM). The sequence of YTHDF2-KD was shown in Additional file 1.

### Western blotting and antibodies

Total proteins were extracted from cultured cells by RIPA buffer. The results showed that the BCA detection method (Beyotime, Nantong, Jiangsu, China) quantitatively detected all proteins. The protein samples separated by PAGE (polyacrylamide gel electrophoresis) were transferred to the PVDF membranes (EMD Millipore, Billerica, Massachusetts, USA). The PVDF membranes were blocked in 5% skim milk for 1 h, then incubated with specific antibodies overnight at 4 °C, and finally incubated with the secondary antibodies for 2 h at room temperature. The membranes were visualized using enhanced chemiluminescence solution (Pierce Biotechnology, Inc. USA). The experimental results were analyzed by ImageJ analysis software. The following antibodies were recorded: anti-GAPDH, anti-FLAG-tag, anti-HIF1α, anti-YTHDF2 (Proteintech, Wuhan, Hubei, China), anti-AKT, anti-Phospho-AKT (Ser473), anti-ERK1/2, anti-Phospho-ERK1/2 (Thr202/Tyr204, Cell Signaling Technologies, Danvers, MA, USA), anti-Phospho-mTOR (Ser2448), anti-mTOR, anti-E-cadherin, anti-N-cadherin, anti-Vimentin, anti-Snail1, anti-METTL3, anti-METTL14.

### Cell treatment

IGF1 (Recombinant human IGF1 protein(Active); cat.no. ab270062; Abcam; 1 ng/ml) was added to NCI-H226 and SK-MES-1 cells which were steadily knockdown YTHDF2 for 24 h to active mTOR/Akt signaling way. The Akt kinase inhibitor (cat. no. ab142088; Abcam; 0.5 µg) was added to NCI-H226 and SK-MES-1 cells which were steadily upregulated YTHDF2 for 24 h to inhibit Akt. After 24 h of treatment, cell proliferation assay, transwell assay and wound-healing assay were carried out.

### Cell proliferation assay

Cell Counting Kit 8 (CCK-8) was used to detect cell proliferation. A total of 2000 cells per well were plated in 96-well plates (Costar; USA). At the indicated time points, 10 μL of CCK-8 reagent (Dojindo) was added to the cells, and the cells were cultured in a humidified incubator (37 ℃, 5% CO2) for another 1 h. Then the optical density at A490 nm was measured by an enzyme-linked immunosorbent assay (ELISA) reader.

### Migration and invasion assays

Transwell chamber assays were cell migration and invasion assays. The transfected cells were plated in the upper chamber of Transwell Matrigel chambers (Collaborative Biomedical Products, USA) at 1.2 × 104 per chamber. For the invasion assay, the lower chamber of the transwell was coated with 150 mg Matrigel (BD Biosciences) diluted with precooled serum‐free DMEM We added serum-free medium and 10% serum-containing medium to the upper and lower layers of the chamber respectively. After 24 h, the invasive cells were fixed, stained, photographed, and quantified. The stained cells were counted in five random fields at × 100 magnification, and the average number was taken.

### The wound-healing assays

The wound-healing assay measured cell migration activity. NCI-H226 and SK-MES-1 cells (4 × 106) were inoculated into six-well plates. After 80% of the cells were fused, 1.3-mm-wide scratched wounds were washed with PBS, and then photographed at 0 and 24 h.

### Animal experiments

The animal experiment was approved by the Animal Research Committee of Jinan Central Hospital Affiliated with Shandong University. Additional file 4 is the detailed original clinical data of 73 LUSC patient.

Thirty-five-day-old male nude mice (athymic BALB/c-nu) were obtained from Shandong University (Jinan, China). The mice were randomly divided into two groups which were inoculated with stable YTHDF2-expressing LUSC cells and the vector LUSC cells. A total of 5 × 10^6^ of the cells were suspended in 0.1 ml of PBS and then injected subcutaneously into the flanks of mice. Tumor size was measured twice a week. The volume formula was: width^2^ × length × π/6. After 5 weeks, well-trained individuals performed physical methods of euthanasia: cervical dislocation on nude mice in a familiar and safe environment, then the xenograft tumor load was isolated, photographed, weighed, and fixed in formalin to perform immunohistochemical staining of YTHDF2.

### Tissue samples and immunohistology

From October 2008 to May 2013, 73 LUSC patients were included in this study at the Department of Jinan Central Hospital Affiliated with Shandong University. We included patients with LUSC diagnosed after complete surgical resection and postsurgical pathology. TNM staging was performed according to the 8th edition of the IASLC Lung Cancer Staging Project [34]. We have obtained informed consent to conduct experiments on human subjects. This study was approved by the Ethics Committee of Jinan Central Hospital Affiliated with Shandong University.

All the LUSC specimens and the adjacent normal lung tissue came from 73 patients. Tissue samples were fixed with a 10% neutral formalin and treated routinely. After dewaxing, inactivation of endogenous peroxidase, and antigen repair, 5% BSA blocking solution was added for 30 min at 37 °C. Then, the sections were incubated with YTHDF2 antibody (1:200, Catalog #A02621-1, BOSTER, Wuhan, China) overnight at 4 °C and secondary antibody (Ready-to-use SABC-POD (rabbit IgG) Kit, Catalog # SA1022, BOSTER, Wuhan, China) at 37 °C for 30 min. The specimens were developed with diaminobenzidine (DAB) and stained with Mayor's hematoxylin at 37 °C for 1 min. Finally, the specimens were analyzed by ImageScope software (Leica) and histochemistry scores were obtained. The median histochemistry score was used to divide patients into two groups.

### Reverse‐transcription quantitative polymerase chain reaction (RT‐qPCR)

Total RNA was extracted from cultured cells using the Trizol reagent (Invitrogen, USA). Total RNA was reversed transcribed into complementary DNA by using HiScript® II 1st Strand cDNA Synthesis Kit (Vazyme). Real‐time PCR was performed using SYBR Green kit and ABI7500 qPCR instrument according to the manufacturer's instructions, with each reaction run in triplicate. The transcription level of the target gene was normalized to β‐actin expression and calculated using 2^−ΔΔCt^ method. The primer for RT-qPCR was listed in Additional file 1.

### Statistical analysis

The experimental data were analyzed by Student's t-test, the chi-squared test, or Fisher’s exact probability test. GraphPad Prism 8.3.0 and the IBM SPSS Statistics 25 were used to perform statistical analyses. Kaplan–Meier survival curves were used in univariate analysis and a Cox predictive risk model was used in multivariate analysis. All the data came from three independent experiments, in triplicate. Moreover, P values less than 0.05 were considered statistically significant.

## Results

### YTHDF2 overexpression promotes cell proliferation and invasion in LUSC.

**Fig. 1:**
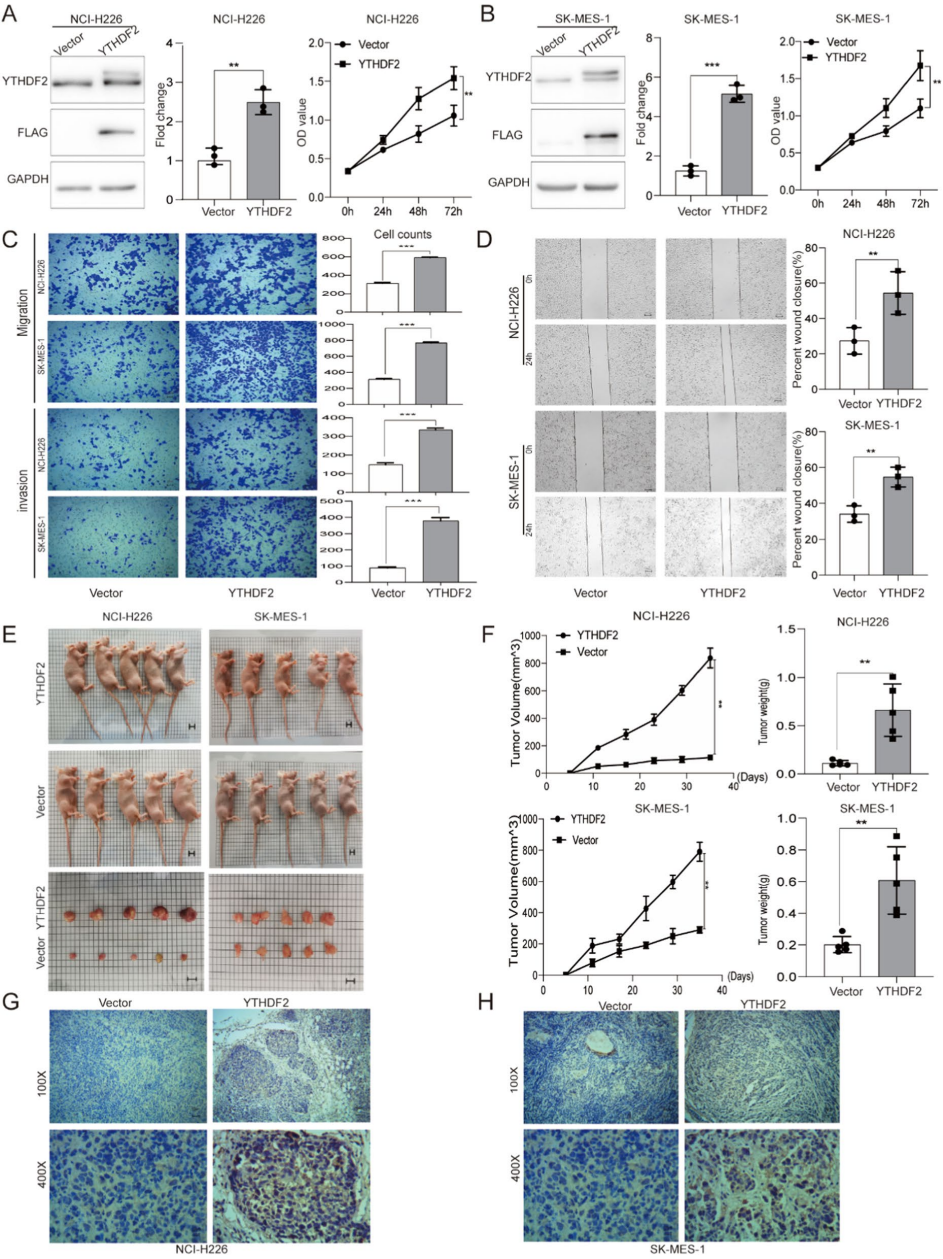
YTHDF2 overexpression promotes cell proliferation and invasion in LUSC. **A** and **B** Representative immunoblot showed that the protein level of YTHDF2 was steadily up-regulated in two LUSC cell lines studied. The CCK8 assay was used to assess cell viability in NCI-H226 and SK-MES-1 cells. **C** and **D** The transwell assay and the wound-healing assay were used to assess the invasion potential and migration ability of NCI-H226 and SK-MES-1 cells. **E** and **F** Tumor size was measured twice a week. After 5 weeks, we dissected tumors from nude mice which had been injected with the indicated stable cell, then measured the tumor size and weight of nude mice injected with the indicated stable cells. **G** and **H** Immunohistochemistry showed the expression level of YTHDF2 from tumors of nude mice injected with the indicated stable cells. Data are represented by the mean ± SD of three independent experiments. *P < 0.05 vs. the vector group

The stable YTHDF2 upregulation models were established in NCI-H226 and SK-MES-1 cells to explore the biological function of YTHDF2 in LUSC. Successful overexpression of YTHDF2 was confirmed at the protein level (Fig. 1A and B). The CCK8 assay was used to confirm that cellular viability dramatically increased in the YTHDF2 group compared with cells carrying the vector only(Fig. 1A and B). Moreover, to explore the effects of YTHDF2 upregulation on cell motility, Transwell assay and wound-healing assay were used to examine the invasion potential and migratory capabilities of LUSC cell lines. The result showed that YTHDF2 overexpression dramatically promoted LUSC cellular invasion abilities and migratory capabilities (Fig. 1C and D). Moreover, a subcutaneous implantation experiment in nude mice was performed to investigate the oncogenic function of YTHDF2 in LUSC. Compared to those bearing the vector only, we showed that stable upregulation of YTHDF2 markedly promoted tumor growth in nude mice, as demonstrated by the significant increase in tumor size and weight (Fig. 1E and F). In the immunohistochemical results, the YTHDF2 expression levels of the xenograft tumors using the indicated stable cells were higher than those carrying the vector only (Fig. 1G and H). Hence, our data suggested that YTHDF2 overexpression plays a critical role in promoting LUSC proliferation and invasion.

### YTHDF2 overexpression upregulates the protein levels of P-AKT and P-mTOR and induces EMT in LUSC cells

**Fig. 2:**
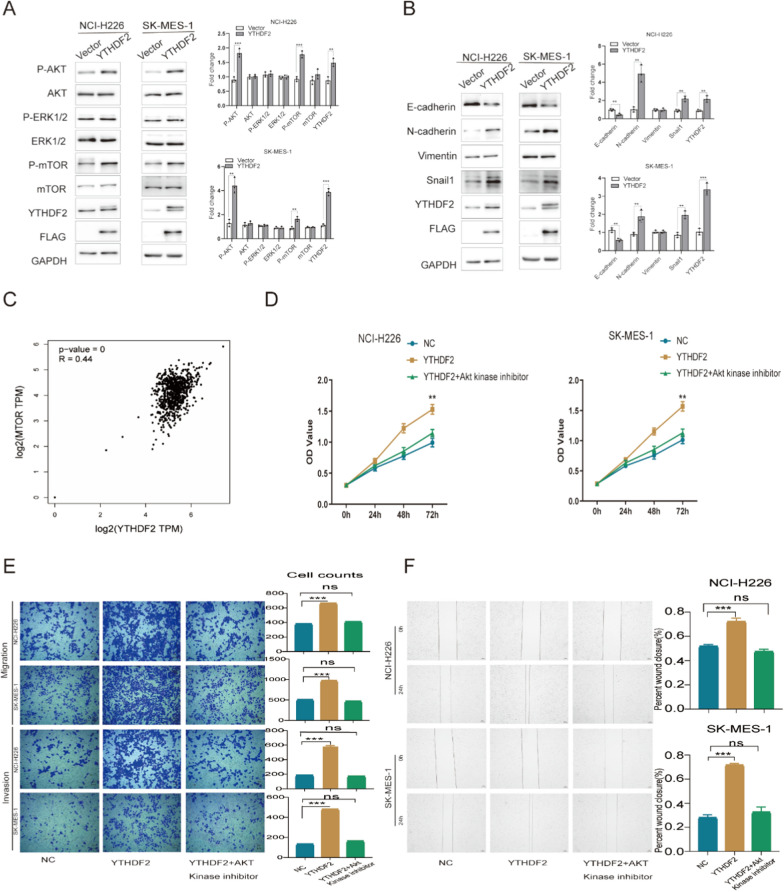
YTHDF2 overexpression upregulates the protein levels of P-AKT and P-mTOR and induces EMT in LUSC cells. **A** The western blot detected the expression of mTOR/AKT-related proteins in NCI-H226 and SK-MES-1 cells. **B** The western blot detected the expression of EMT-related proteins in NCI-H226 and SK-MES-1 cells. **C** Using the Pearson correlation statistics, we examine the pairwise gene correlation analysis between YTHDF2 and mTOR in LUSC through GEPIA’ TCGA and GTEx expression data. **D** and **E** and **F** YTHDF2 overexpression resulted in significant promotion of cellular proliferation, invasion and migration in NCI-H226 and SK-MES-1 cells, while AKT Kinase inhibitor eliminated the promotion of YTHDF2 overexpression. Data are represented by the mean ± SD of three independent experiments. *P < 0.05 vs. the vector group

We investigated whether YTHDF2 overexpression activates key signaling pathways in LUSC cells, such as the ERK/MAPK and mTOR/AKT signaling pathways known to play a role in tumor proliferation and survival. The results showed that compared with cells carrying vector-only, the phosphorylation of AKT and mTOR in the YTHDF2 group was markedly increased, but not of ERK (Fig. 2A). YTHDF2 overexpression also led to down-regulation of E-cadherin and up-regulation of N-cadherin and Snail1 at protein levels in NCI-H226 and SK-MES-1 cells (Fig. 2B). In addition, the GEPIA (http://gepia.cancer-pku.cn/) expression data further confirm YTHDF2 expression is positively correlated with mTOR in LUSC (Fig. 2C). We found YTHDF2 overexpression induces high expression of P-AKT (Ser473) and P-mTOR and promotes cell proliferation and invasion in LUSC cells. To further determine whether YTHDF2 overexpression promotes cell proliferation and invasion in LUSC via the mTOR/AKT signaling pathway, AKT Kinase inhibitor was used in YTHDF2 overexpressed group of cells. The CCK8 assay (Fig. 2D), the transwell assay(Fig. 2E) and the wound-healing assay(Fig. 2F) were used to evaluate cell proliferation, invasion and migration ability. We found that YTHDF2 overexpression enhanced cellular proliferation, invasion and migration ability compared to the control group in both NCI-H226 and SK-MES-1, while the knockdown of Akt by Akt kinase inhibitor (Akt-1/2, ab142088; Abcam) abolished the effects of YTHDF2 overexpression on promotion cellular proliferation and invasion in LUSC cells. Therefore, we posit that YTHDF2 overexpression activates the mTOR/AKT pathway to promote cell proliferation and invasion, which certainly needs to be further confirmed by the following experiments.

### YTHDF2 knockdown inhibits cell proliferation and invasion in LUSC

**Fig. 3:**
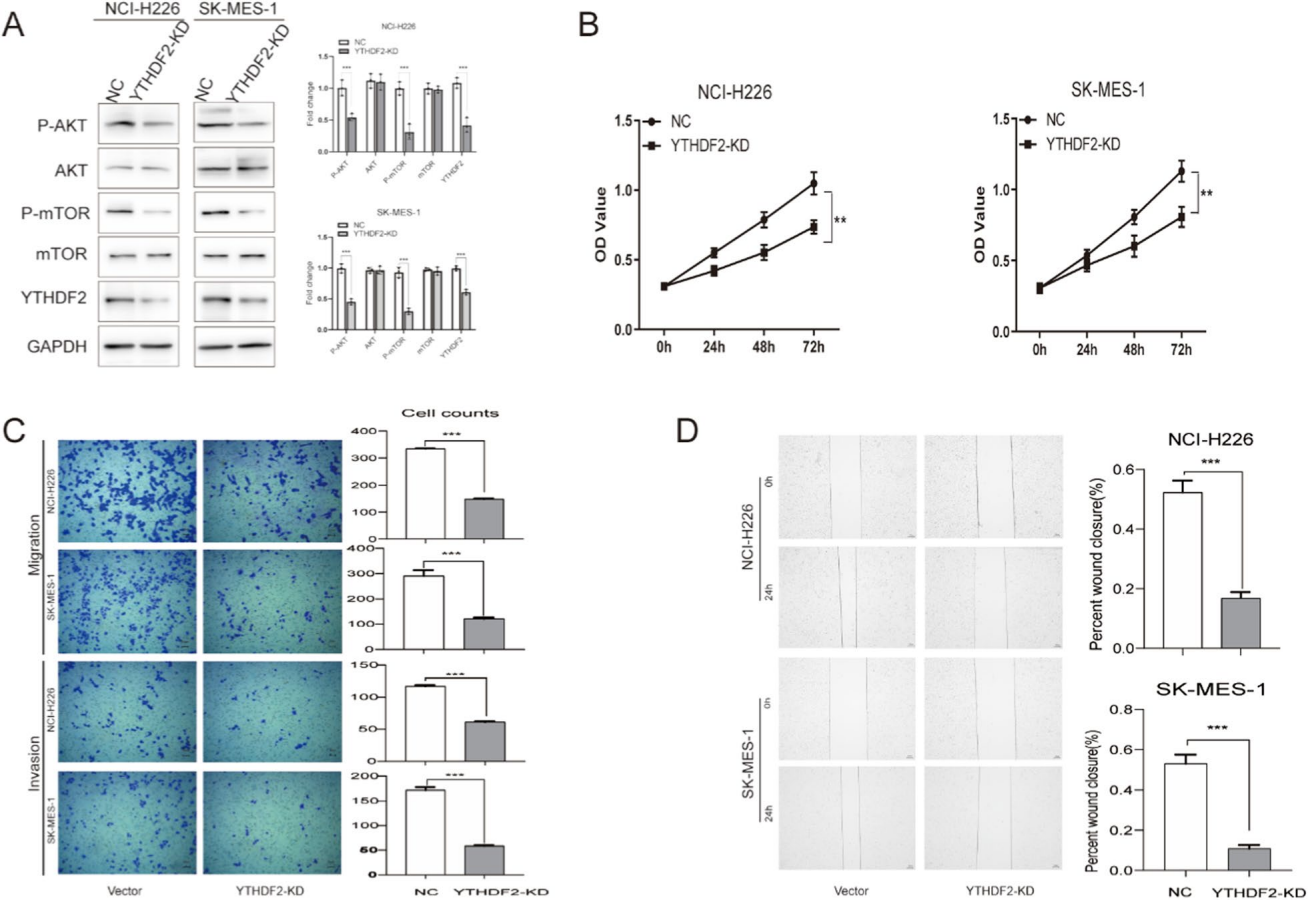
YTHDF2 knockdown inhibits cell proliferation and invasion in LUSC. **A** The western blot analyzed the expression of mTOR/AKT-related proteins in NCI-H226 and SK-MES-1 cells. **B** and **C** NCI-H226 and SK-MES-1 had YTHDF2 knocked out, resulting in significant inhibition of cell proliferation, migration and invasion. **D** YTHDF2 knockdown significantly inhibits cell migration viability in NCI-H226 and SK-MES-1 cells by the wound-healing assay. Data are represented by the mean ± SD of three independent experiments. *P < 0.05 vs. the vector group

We targeted the knockdown of the endogenous expression of YTHDF2 in NCI-H226 and SK-MES-1 cells. The western blot assay showed that the endogenous expression of YTHDF2 was effectively knocked down in NCI-H226 and SK-MES-1 cells, while P-AKT (S473) and P-mTOR were inhibited (Fig. 3A). To verify the potential function of YTHDF2 in LUSC cells, we performed the cell proliferation, invasion, and migration associated assays. The CCK8 assay revealed that YTHDF2 knockdown reduced the cell proliferation rate in LUSC cells (Fig. 3B). The transwell assay and wound-healing assay showed YTHDF2 knockdown inhibited the cell migration and invasion rate (Fig. 3C and D), Take together, YTHDF2 knockdown inhibits the protein levels of P-AKT and P-mTOR, and weaken the ability of cellular proliferation and invasion in LUSC cells.

### YTHDF2, as a tumor promoter, may lead to a poor prognosis for LUSC patients.

**Fig. 4:**
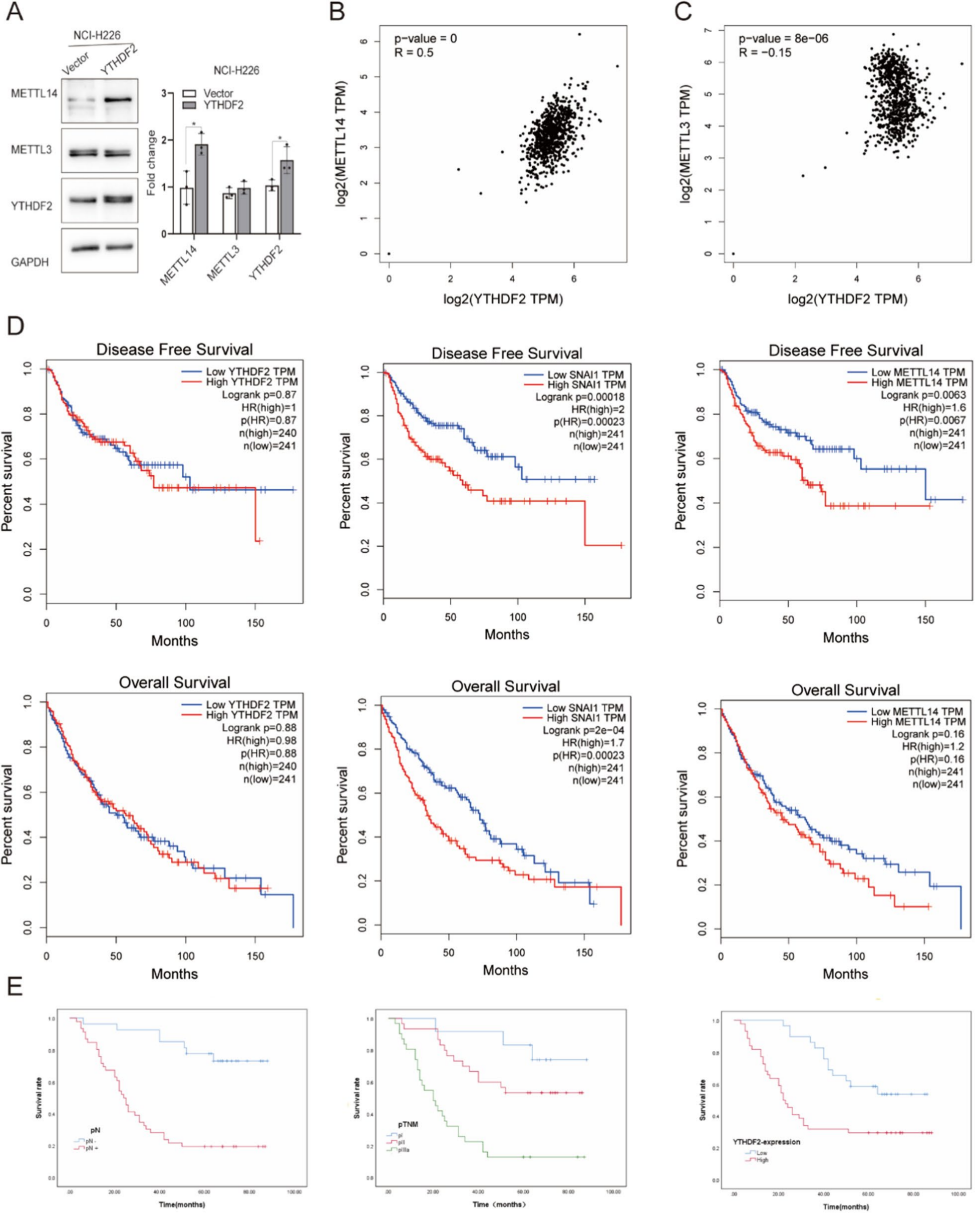
YTHDF2, as a tumor promoter, may lead to a poor prognosis for LUSC patients. **A** Representative immunoblot showed YTHDF2 overexpression to promote METTL14 upregulation in NCI-H226, not of METTL3. Data are represented by the mean ± SD of three independent experiments. *P < 0.05 vs. the vector group. **B** and **C** Using the Pearson correlation statistics, we examine the pairwise gene correlation analysis between YTHDF2 and METTL14, YTHDF2 and METTL3 by TCGA and GTEx expression data of GEPIA. **D** By a log-rank test for the overall survival (OS) and disease-free survival (DFS) analysis in LUSC, we respectively investigate gene YTHDF2, METTL14, and Snail by the ‘Survival’ tab of GEPIA. **E** In the immunohistochemical staining results, the overall survival of LUSC patients with pN, pTNM, and YTHDF2 expression was analyzed by A Kaplan–Meier analysis

Recently, an increasing number of studies have examined the correlation analysis between writer proteins and reader proteins [35, 36]. Our data suggested that the overexpression of YTHDF2 directly affected the expression level of METTL14 at the protein level (Fig. 4A). Using the GEPIA (http://gepia.cancer-pku.cn/), we also found a marked correlation between the expression of YTHDF2 and METTL14, but not METTL3 in LUSC (Fig. 4B and C). Therefore, we speculated that YTHDF2 cooperating with METTL14 may be involved in LUSC.

Using the GEPIA (http://gepia.cancer-pku.cn/) to detect the overall survival (OS) and the disease-free survival (DFS) of genes (YTHDF2, Snail, METTL14), the results showed that LUSC patients at the mRNA level with increased expression of Snail had poor DFS and OS, but all not of YTHDF2, while the upregulation of METTL14 only lessened DFS (Fig. 4D). In the immunohistochemical staining results, YTHDF2 protein expression levels in lung squamous cell carcinoma tissues were markedly higher than those in the corresponding normal lung tissues (Table 1 and Additional file 1: Fig. S1). We further explored the correlation between YTHDF2 expression and clinicopathological characteristics. The 5-year survival rate of the 73 LUSC patients accounted for 39.7%. Table 2 shows that the YTHDF2 upregulation was markedly correlated with pN (pN– 37.0%, pN + 73.9%; P = 0.002) and pTNM stage (pI 50.0%, pII 43.3%, pIIIa 80.6%; P = 0.007). According to the log-rank test with univariate analysis, the 5-year survival rate of LUSC patients was closely related to pN (P = 0.001), pTNM stage (P = 0.01), and high expression of YTHDF2 (P = 0.002, Table 3). Ultimately, Cox regression with multiple analyses showed that pN and YTHDF2 expression acted as independent factors affecting the 5-year survival rate (Table 4). Consistently, these data showed that YTHDF2, which induced the high expression of METTL14 and Snail, may lead to a worse prognosis for LUSC patients.

**Table 1:** YTHDF2 expression in LUSC compared with para-carcinoma tissue

Group	n	YTHDF2 expression		P
		Low (n%)	High (n%)	
LUSC	73	29 (39.7)	44(60.3)	0.000^***^
para-carcinoma	73	58 (79.5)	15(20.5)	

P-value: chi-squared test

**Table 2:** Relationship between YTHDF2 and clinicopathological factors of LUSC patients

Clinicopathological parameters	N	YTHDF2 Low (n = 29)	YTHDF2 High (n = 44)	P
Gender				0.298
Male	66	28	38	
Female	7	1	6	
Age				0.337
< 60	23	11	12	
≥ 60	50	18	32	
Differentiation				0.550
Good	3	0	3	
Moderate	53	22	31	
Poor	17	7	10	
pT classification				0.901
pT1	12	4	8	
pT2	32	13	19	
pT3	29	12	17	
pN				0.002
−	27	17	10	0.007
+	46	12	34	
pTNM stage				
pI	12	6	6	
pII	30	17	13	
pIIIa	31	6	25	

P-value: chi-squared test, Fisher’s exact probability test. pT classification, tumor size; pN, lymph node metastasis and pTNM stage, tumor stage

**Table 3:** Results of univariate analysis concerning 5-year survival of the LUSC patients

Clinical features	Patients (N = 73)	5-year survival (%)		P
		patients	Rate (%)	
Gender				0.711
Male	66	26	39.4	
Female	7	3	42.9	
Age				0.451
< 60	23	10	43.5	
≥ 60	50	19	38.0	
Differentiation				0.928
Good	3	2	66.7	
Moderate	53	21	39.6	
Poor	17	6	35.3	
pT classification				0.465
pT1	12	6	50.0	
pT2	32	13	40.6	
pT3	29	10	34.5	
pN				0.001
−	27	19	70.4	
+	46	10	21.7	
pTNM stage				0.001
pI	12	9	75.0	
pII	30	15	50.0	
pIIIa	31	5	16.1	
YTHDF2 expression				0.002
Low	29	16	55.2	
High	44	13	29.5	

P-Log-rank test; pT classification, tumor size; pN, lymph node metastasis and pTNM stage, tumor stage

**Table 4:** Results of Cox multivariate regression 5-year survival rate of the LUSC patients

Clinical features	B	SE	Wald	P	HR	95.0%CI for HR
Differentiation	0.616	0.343	3.219	0.073	1.851	0.945–3.626
pT classification	0.274	0.250	1.195	0.274	1.315	0.805–2.147
pN	1.669	0.650	6.588	0.010	5.309	1.484–18.997
pTNM stage	0.254	0.403	0.397	0.528	1.289	0.585–2.840
YTHDF2expression	0.781	0.355	4.856	0.028	2.184	1.090–4.377

B, regression coefficient; SE, standard error; Wald, Wald value; HR, hazard ratio; CI, confidence interval; pT classification, tumor size; pN, lymph node metastasis and pTNM stage, tumor stage

### Hypoxia specifically induces YTHDF2 overexpression to activate the mTOR/AKT axis in LUSC cells

Hypoxia induces hypoxia-inducible factor-1α (HIF-1α), which is mediated by a proline hydroxylase and has emerged as a crucial factor. Moreover, hypoxia is associated with poor prognosis and resistance to radiation and chemotherapy [37].

**Fig. 5:**
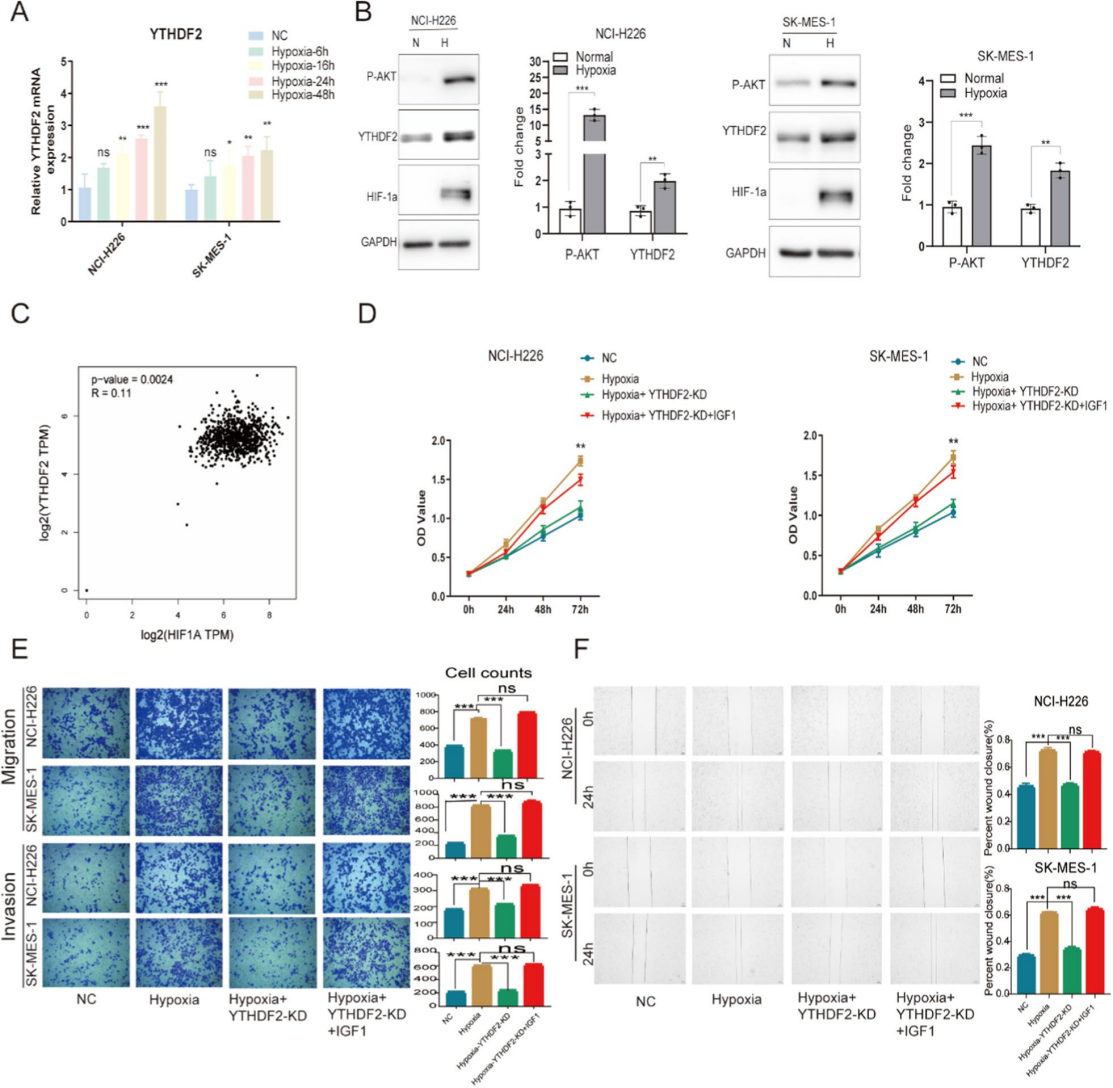
Hypoxia specifically induces YTHDF2 overexpression to activate the mTOR/AKT axis in LUSC cells. **A** RT-qPCR was used to investigate the alterations of mRNA levels of YTHDF2 when LUSC cells were exposed to 1% O2 for 6 h, 16 h, 24 h, 48 h, respectively. **B** NCI-H226 and SK-MES-1 cells were exposed with 20% O2 or 1% O2 (1% O2, 5%CO2, 94% N2) for 24 h. The protein levels of HIF-1α, YTHDF2, and P-AKT (be phosphorylated at serine 473) were analyzed by western blot. **C** Using the Pearson correlation statistics, we examine the pairwise gene correlation analysis between HIF-1α and YTHDF2 by TCGA and GTEx expression data of GEPIA. **D** The cellular growth was analyzed by CCK8 assay at different time points. **E** The cellular invasion and migration growths were analyzed by transwell assay. **F** The migration viability was analyzed by wound-healing assay. Data are represented by the mean ± SD of three independent experiments. *P < 0.05 vs. the vector group

We assume whether the mTOR/AKT axis is the mediator for YTHDF2 responding to hypoxia-induced cellular proliferation and invasion. We first confirmed that the mRNA level of YTHDF2 was upregulated in a time-dependent manner under hypoxia by Real-time RT-qPCR (Fig. 5A). Additional files 2 and 3 are original experimental results of RT-PCR ,which are used to investigate the mRNA levels of YTHDF2 upon hypoxia in a time-dependent manner. By western blot, the expression of HIF-1α was used to validate the hypoxic response. We further confirmed that the protein levels of YTHDF2 and P-AKT (Ser473) were increased in hypoxia-mediated (Fig. 5B). Meanwhile, we employed bioinformatics-based screening to explore the association of HIF-1α and YTHDF2 in human lungs. LUSC data mining of the GEPIA(http://gepia.cancer-pku.cn/) showed that HIF-1α expression was positively correlated with YTHDF2 (Fig. 5C). CCK8 assay, transwell assay, and wound-healing assay showed that hypoxia significantly promoted cellular proliferation, migration, and invasion in LUSC cells(Fig. 5D, E and F). To further determine whether hypoxia mediates the regulation of cellular proliferation, migration, and invasion by YTHDF2 upregulation activating AKT/mTOR axis, we knocked out YTHDF2 in NCI-H226 and SK-MES-1 under hypoxia, significantly inhibiting cellular proliferation, migration, invasion compared with cell only under hypoxia. In addition, IGF1 rescued the inhibition of cellular proliferation invasion migration growth of YTHDF2 knockdown under hypoxia in LUSC cells(Fig. 5D, E and F). Therefore, we found that hypoxia specifically induces YTHDF2 overexpression promotes LUSC progression by activating the mTOR/AKT axis.

## Discussion

In this study, we suggested that YTHDF2 upregulation was significantly induced by hypoxia in LUSC cells. Overexpression of YTHDF2 positively activates the mTOR/AKT pathway and regulates the progression of EMT which may act as a tumor promoter to induce LUSC cell proliferation and invasion.

Generally, it has been reported that m6A modification is associated with tumorigenesis. A better explanation of the molecular mechanisms of a complete m6A modification process requires the cooperation of m6A writer genes, erasers genes, and readers genes, rather than a single isolated gene. For example, m6A modification mediated by the cooperation of METTL14, ALKBH5, and YTHDF3 was reported to influence the cell cycle, induce the progression of EMT, and contribute to angiogenesis of cancer cells in breast cancer [37]. YTHDF1-mediated the translation of Snail was verified, as a portion of EMT was altered by deletion of METTL3 in liver patients [30]. In bladder cancer, the mutual interaction between METTL3 and YTHDF2 induced the degradation of SETD7 and KLF4 mRNA in the proliferation and metastasis process [32]. Moreover, the methyltransferase METTL3 was discovered to regulate the degradation of SOCS2 mRNA, enhancing the progression of liver cancer in a YTHDF2-mediated m6A-dependent manner [36]. Here, we also demonstrated that YTHDF2 was positively related to METTL14 and cooperated with METTL14 in LUSC. However, how this mutual collaboration between METTL14 and YTHDF2 activates the oncogenic signaling pathway in LUSC is largely unknown. We will address the unknown mechanism in our next study.

In our study, the YTHDF2 protein expression level was markedly higher in human LUSC tissues than in normal tissues. Cell proliferation was significantly enhanced in YTHDF2-overexpression cells compared with control cells. Moreover, YTHDF2 upregulation promoted tumor growth and increased tumor volume in vivo compared with control cells. The mechanism underlying YTHDF2-mediated LUSC tumorigenesis was also investigated. The results indicated that compared with control cells, AKT and mTOR phosphorylation was significantly increased following YTHDF2 overexpression, which is crucial for tumor progression. Based on the results, it was hypothesized that YTHDF2 may promote LUSC cell proliferation by activating the AKT/mTOR signaling pathway which is known to play a pivotal role in multiple types of cancer, such as breast cancer and ovarian carcinoma [38, 39]. To the best of our knowledge, the present study is the first study to demonstrate the role of YTHDF2 in LUSC cell proliferation. However, our present study failed to analyze AKT and mTOR expression levels by immunohistochemistry.

Our study was not without limits: for instance, there are reports that YTHDF2 regulates m6A-modified mRNA degradation, which is seemingly contrary to our study. One possible explanation is that YTHDF2 decays the m6A-modified mRNA of tumor suppressor genes, thus promoting cell growth. Another possible explanation is that YTHDF2-mediated mRNA decay might not be the only mechanism underlying m6A function in cancer progression. In addition, one report suggests that PI3K-AKT signaling is involved in promoting the EMT process via the mTOR or MAPK cascade [40], and another report indicates that the m6A modification is associated with the EMT progression and cancer metastasis that is induced by YTHDF1-mediated Snail translation in liver patients [38]. Therefore, the existence of these two signaling axes remains to be verified in LUSC, and whether m6A mediates E-cadherin expression via other factors/pathways also deserves further exploration. In future studies, we will contribute to addressing these unresolved limitations.

In summary, the present study indicated that hypoxia-mediated YTHDF2 overexpression activates the mTOR/AKT signaling pathway to promote LUSC cell proliferation and invasion. These results may improve the current understanding of the mechanism underlying the biological role of YTHDF2 during tumor development and might provide a potential therapeutic target for LUSC.

## Supplementary Information

**Additional file 1: Figure S1.** Immunohistochemical staining of LUSC tissue sections demonstrating YTHDF2. (A) The corresponding normal lung tissue specimen with low expression of YTHDF2. (B) Lung squamous cell carcinoma specimen with high expression of YTHDF2.

**Additional file 2.** Hypoxia-RT-qPCR-NCI-H226.

**Additional file 3.** Hypoxia-RT-qPCR-SK-MES-1.

**Additional file 4.** The clinicopathological characteristics of LUSC.

## Data Availability

All data are included in this article.

## Abbreviations

m6A: N6-methyladenosine
YTHDF2: YTH domain family 2
LUSC: Lung squamous cell carcinoma
HIF-1α: Hypoxia-inducible factor-1α
LUAD: Lung adenocarcinoma
NSCLC: Non-small cell lung cancer
miRNA: MicroRNA
EMT: The epithelial-mesenchymal transition
ATCC: The American Tissue Culture Collection
PAGE: Polyacrylamide gel electrophoresis
CCK-8: Cell Counting Kit 8
ELISA: Enzyme-linked immunosorbent assay
DAB: Diaminobenzidine
OS: The overall survival
DFS: The disease-free survival
