# Correction to: Hypoxia-mediated YTHDF2 overexpression promotes lung squamous cell carcinoma progression by activation of the mTOR/AKT axis

**DOI:** 10.1186/s12935-023-03030-5

**Published:** 2023-08-26

**Authors:** Peng Xu, Kang Hu, Ping Zhang, Zhi-Gang Sun, Nan Zhang

**Affiliations:** 1 Cheeloo College of Medicine, Shandong University, Jinan, 250013 Shangdong PR China; 2 School of Clinical Medicine, Weifang Medical University, Weifang, 261053 Shangdong PR China; 3 Department of Thoracic Surgery, Cheeloo College of Medicine, Jinan Central Hospital, Shandong University, Jinan, 250013 Shandong PR China; 4 Department of Oncology, Cheeloo College of Medicine, Jinan Central Hospital, Shandong University, Jinan, 250013 Shandong PR China

**Correction to: Cancer Cell International (2022) 22:13**

10.1186/s12935-021-02368-y

Following the publication of the original article [1], we were notified of an error in Fig. 1H. The corrected Fig. 1H can be found below.

**Fig. 1:**
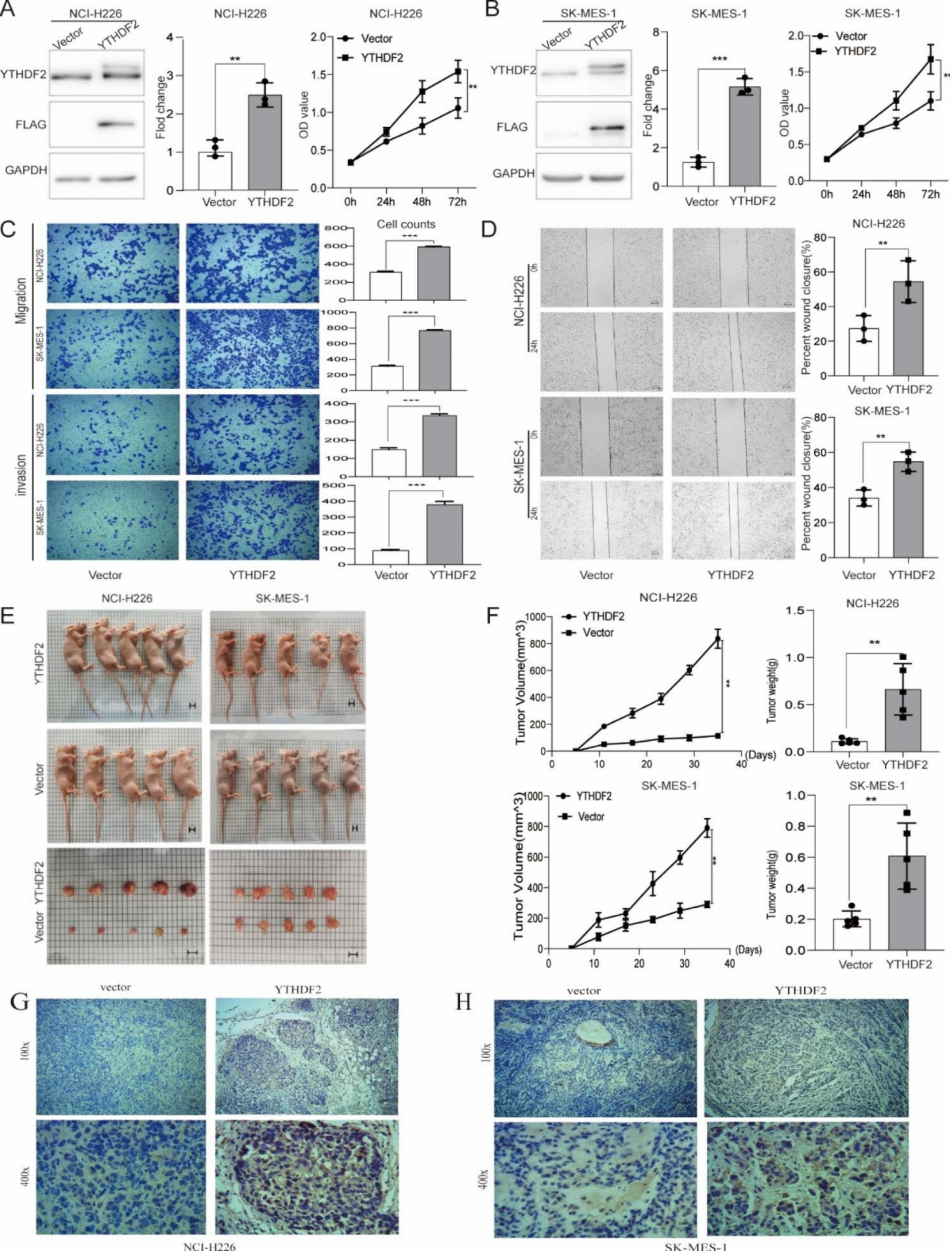
YTHDF2 overexpression promotes cell proliferation and invasion in LUSC. **A** and **B** Representative immunoblot showed that the protein level of YTHDF2 was steadily up‑regulated in two LUSC cell lines studied. The CCK8 assay was used to assess cell viability in NCI‑H226 and SK‑MES‑1 cells. **C** and **D** The transwell assay and the wound‑healing assay were used to assess the invasion potential and migration ability of NCI‑H226 and SK‑MES‑1 cells. **E** and **F** Tumor size was measured twice a week. After 5 weeks, we dissected tumors from nude mice which had been injected with the indicated stable cell, then measured the tumor size and weight of nude mice injected with the indicated stable cells. **G** and **H** Immunohistochemistry showed the expression level of YTHDF2 from tumors of nude mice injected with the indicated stable cells. Data are represented by the mean ± SD of three independent experiments. *P < 0.05 vs. the vector group
