# Supplementary material for: Hypoxia-mediated YTHDF2 overexpression promotes lung squamous cell carcinoma progression by activation of the mTOR/AKT axis
Source: Cancer Cell Int. 2022 Jan 7;22:13. doi: 10.1186/s12935-021-02368-y (PMC8742419; doi:10.1186/s12935-021-02368-y)
Supplement: Supplementary file 1 — Additional file 1: Figure S1. Immunohistochemical staining of LUSC tissue sections demonstrating YTHDF2. (A) The corresponding normal lung tissue specimen with low expression of YTHDF2. (B) Lung squamous cell carcinoma specimen with high expression of YTHDF2. [file 12935_2021_2368_MOESM1_ESM.docx]

1. The primer used to amplify the gene of YTHDF2 is as follows**.**

|  | qRT-PCR Primers | |
| --- | --- | --- |
| Gene | Forward Sequence(5′-3′) | Reverse sequence(5′-3′) |
| YTHDF2 | TAGCCAACTGCGACACATTC | CACGACCTTGACGTTCCTTT |

2. Plasmid construction

**(1)YTHDF2-KD**

sh-1-F

GATCCCCGCTACTCTGAGGACGATATTCTTCAAGAGAGAATATCGTCCTCAGAGTAGCTTTTTA

sh-1-R

AGCTTAAAAAGCTACTCTGAGGACGATATTCTCTCTTGAAGAATATCGTCCTCAGAGTAGCGGG

1. YTHDF2

3.IGF1(Recombinant human IGF1 protein(Active)) (ab270062) (abcam, United Kingdom)

Sequence:

GP ETLCGAELVD ALQFVCGDRG FYFNKPTGYG SSSRRAPQTG IVDECCFRSC DLRRLEMYCA PLKPAKSA


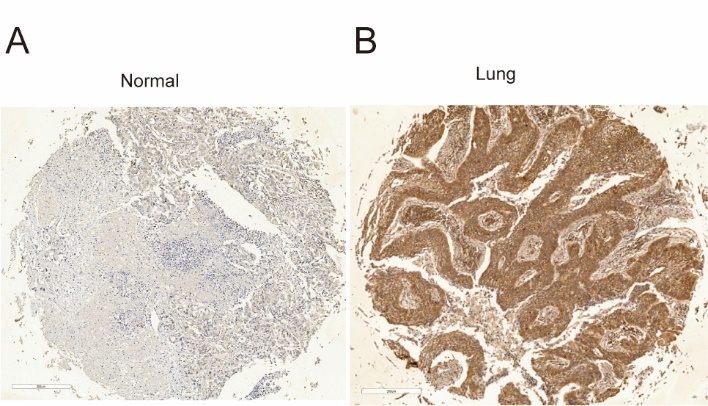


**Figure S1.** Immunohistochemical staining of LUSC tissue sections demonstrating YTHDF2. (A) The corresponding normal lung tissue specimen with low expression of YTHDF2. (B) Lung squamous cell carcinoma specimen with high expression of YTHDF2.
